# Genome-wide association study for Chagas Cardiomyopathy identify a new risk locus on chromosome 18 associated with an immune-related protein and transcriptional signature

**DOI:** 10.1371/journal.pntd.0010725

**Published:** 2022-10-10

**Authors:** Ester Cerdeira Sabino, Lucas Augusto Moysés Franco, Gabriela Venturini, Mariliza Velho Rodrigues, Emanuelle Marques, Lea Campos de Oliveira-da Silva, Larissa Natany Almeida Martins, Ariela Mota Ferreira, Paulo Emílio Clementino Almeida, Felipe Dias Da Silva, Sâmara Fernandes Leite, Maria do Carmo Pereira Nunes, Desiree Sant’Ana Haikal, Claudia Di Lorenzo Oliveira, Clareci Silva Cardoso, Jonathan G. Seidman, Christine E. Seidman, Juan P. Casas, Antonio Luiz Pinho Ribeiro, Jose E. Krieger, Alexandre C. Pereira

**Affiliations:** 1 Departamento de Moléstias Infecciosas e Parasitárias, Hospital das Clínicas da Faculdade de Medicina da Universidade de São Paulo (FMUSP), São Paulo, Brazil; 2 Laboratório de Parasitologia Médica (LIM-46), Hospital das Clínicas da Faculdade de Medicina da Universidade de São Paulo (FMUSP), São Paulo, Brazil; 3 Laboratorio de Genetica e Cardiologia Molecular, Instituto do Coracao (InCor), Faculdade de Medicina da Universidade de São Paulo, São Paulo, Brazila; 4 Genetics Department, Harvard Medical School, Boston, Massachusetts, United States of America; 5 Department of Statistics, Instituto de Ciência Exatas, Universidade Federal de Minas Gerais, Belo Horizonte, Brazil; 6 Universidade Estadual de Montes Claros, Montes Claros, Minas Gerais, Brazil; 7 Health Science Program, Universidade Estadual de Montes Claros, Montes Claros, Brazil; 8 Faculdade de Medicina, Universidade Federal de Minas Gerais, Belo Horizonte, Brazil; 9 Federal University of São João del-Rei, Divinópolis, Brazil; 10 Cardiovascular Division, Brigham and Women’s Hospital, Boston, Massachusetts, United States of America; 11 Howard Hughes Medical Institute, Chevy Chase, Maryland, United States of America; 12 Massachusetts Veterans Epidemiology Research and Information Center, Veterans Affairs Boston Healthcare System, Boston, Massachusetts, United States of America; 13 Division of Aging, Brigham and Women’s Hospital, Harvard Medical School, Boston, Massachusetts, United States of America; 14 Telehealth Center, Hospital das Clínicas, Universidade Federal de Minas Gerais, Belo Horizonte, Brazil; University of Texas at El Paso, UNITED STATES

## Abstract

**Background:**

Chronic Chagas Cardiomyopathy (CCC) usually develops between 10 and 20 years after the first parasitic infection and is one of the leading causes of end-stage heart failure in Latin America. Despite the great inter-individual variability in CCC susceptibility (only 30% of infected individuals ever present CCC), there are no known predictors for disease development in those chronically infected.

**Methodology/Principal findings:**

We describe a new susceptibility locus for CCC through a GWAS analysis in the SaMi-Trop cohort, a population-based study conducted in a Chagas endemic region from Brazil. This locus was also associated with CCC in the REDS II Study. The newly identified locus (rs34238187, OR 0.73, p-value 2.03 x 10^−9^) spans a haplotype of approximately 30Kb on chromosome 18 (chr18: 5028302–5057621) and is also associated with 80 different traits, most of them blood protein traits significantly enriched for immune-related biological pathways. Hi-C data show that the newly associated locus is able to interact with chromatin sites as far as 10Mb on chromosome 18 in a number of different cell types and tissues. Finally, we were able to confirm, at the tissue transcriptional level, the immune-associated blood protein signature using a multi-tissue differential gene expression and enrichment analysis.

**Conclusions/Significance:**

We suggest that the newly identified locus impacts CCC risk among *T cruzi* infected individuals through the modulation of a downstream transcriptional and protein signature associated with host-parasite immune response. Functional characterization of the novel risk locus is warranted.

## Author summary

Chronic Chagas Cardiomyopathy (CCC) usually develops between 10 and 20 years after the first parasitic infection and is one of the leading causes of end-stage heart failure in Latin America. Despite the great inter-individual variability in CCC susceptibility, there are no known predictors for disease development in those chronically infected. We describe a new susceptibility locus for CCC through a GWAS analysis in the SaMi-Trop cohort, a population-based study conducted in a Chagas endemic region from Brazil. The newly identified locus on chromosome 18 is also associated with 80 different traits, most of them blood protein traits significantly enriched for immune-related biological pathways. Finally, we were able to confirm, at the tissue transcriptional level, the immune-associated blood protein signature using a multi-tissue differential gene expression and enrichment analysis. We suggest that the newly identified locus impacts CCC risk among *T cruzi* infected individuals through the modulation of a downstream transcriptional and protein signature associated with host-parasite immune response.

## Introduction

An estimated 6 to 9 million people worldwide are currently infected with *Trypanosoma cruzi* and approximately 70 million are at risk of infection [1]. Each year the disease is responsible for 37,000 new cases, 13,000 deaths and 649,000 disability-adjusted life years (DALYs) [2]. Although *T*. *cruzi* is endemic in Latin America, infected immigrants live around the world, including ~300,000 in the U.S. alone [3]. Thirty percent of infected individuals develop Chronic Chagas Cardiomyopathy (CCC), an enigmatic disease characterized by ventricular dilation and reduced cardiac function that cause arrhythmias, thromboembolisms, heart failure, stroke, and premature death [4]. There are no predictors to who, among infected individuals, will develop late-stage cardiac disease. Likewise, there are no cures for CCC and a large-scale trypanocidal therapy study showed no improvement of chronic benznidazole treatment on clinical deterioration [5]. In fact, at least 1 million Latin American people carrying the parasite will die unless new scientific and political breakthroughs occur.

The great majority of acute *T*. *cruzi* infections are unapparent and most symptomatic patients present with minor clinical manifestations. Most untreated acute cases evolve into the indeterminate stage of chronic Chagas disease (seropositive but no sign of the cardiac or digestive forms of the disease). However, some individuals will develop cardiomyopathy and/or the mega-syndromes generally approximately 10 to 20 years after infection in a slow but progressive fashion [4].

Unfortunately, there are no good clinical or demographic predictors of who, among infected individuals, will develop the more severe cardiomyopathy form. Here we hypothesize that common genetic variation may be able to explain, at least in part, why 30% of chronically infected Chagas patients will develop end-stage heart damage. To test this hypothesis, we have conducted a GWAS and meta-analysis using data from two Brazilian cohorts aimed at identifying determinants of CCC development.

## Materials and methods

### Ethics statement

The local ethics committee of the Hospital das Clínicas, University of São Paulo (CEP 042/12), approved the study protocol, in accordance with the Declaration of Helsinki. All participants in this study have signed a written statement for formal consent.

### Study sample–SaMi-Trop

The study sample belongs to the SaMi-Trop Study. For the present analysis, we used samples from the 3,398 participants enrolled in the study.

The SaMi-Trop study was established as a prospective cohort of patients with CCC with the aim to evaluate if a clinical prediction rule for CCC based on ECG, brain natriuretic peptide (BNP) levels, and other biomarkers could be useful in clinical practice [6]. Initial enrollment occurred between 2013 and 2014 and ascertained 1,959 individuals. During the first follow-up visit (between 2015 and 2019) another 1,439 individuals were enrolled in the study.

After signing an informed consent, a sample of whole-blood was collected for biobanking and used for genomic DNA extraction.

Participants responded to a detailed questionnaire aimed at collecting demographic, risk factors, symptoms, and prior clinical information. All eligible participants were tested for the presence of anti-T. cruzi antibodies using chemiluminescent microparticle immunoassay. Negative results were confirmed by two other enzyme immunoassay (EIA) presenting different antigens. The final sample consists of individuals confirmed to be seropositive. In addition, a resting 12-lead ECG was recorded using an ECG PC machine (TEB, São Paulo, Brazil). The ECG recordings were sent electronically to the Telehealth system and read by a trained cardiologist; the written report was subsequently returned to the patient’s physician. For research purposes, ECGs were also automatically analyzed using the University of Glasgow ECG analysis programme (release 28.5, issued on January 2014) and reviewed by trained cardiologists to ensure quality control. ECGs were classified using the Minnesota Code criteria using variables derived from the median complex of the Glasgow University software measurement matrix.

### Chagas Cardiomyopathy definition in the SaMi-Trop cohort

We used a previously validated definition of Chagas Cardiomyopathy for the Brazilian population [7]. Briefly, we have considered all participants with major or minor typical ECG abnormalities to have CCC. Major typical ECG abnormalities were: Typical RBB block (with or without LAHB), complete intraventricular block, frequent ventricular premature beats, major primary isolated ST segment or T-wave abnormalities, atrial fibrillation or flutter, sinus bradycardia (HR < 40 bpm), major atrioventricular conduction abnormalities (2nd or 3rd degree) and pacemaker use. Individuals with minor ECG abnormalities were also considered with CCC for the present analysis. The present analysis used only individuals without missing data. Therefore, the final numbers of classified individuals were 2964, with 581 in the group of participants without CCC and 2,383 individuals with CCC.

### SaMi-Trop SNP genotyping and imputation

Genomic DNA extraction has been previously described [8]. SaMi-Trop DNA samples were genotyped using two different genotyping arrays: Axiom_PMRA.r3 array (N = 2,606) or the Axiom_sarscov array (N = 792) (ThermoFisher, Waltham, USA) and genotypes annotated using the array specific annotation file provided at the ThermoFisher website. Genotype calling was performed using Affymetrix Power Tools [9]. Initial VCF file contained 701,985 (for the PMRA array) and 803,863 (for the sarscov array) variants before quality control filtering.

Imputation was performed using the Haplotype Reference Consortium Michigan Imputation Server using the TOPMED reference haplotype panel as reference [10] (for mixed samples). More specifically, the Michigan Imputation Server [11] used Minimac4 to conduct imputation on 586,589 SNPs remaining after data quality control for samples genotyped using the PMRA array and 669,135 for samples genotyped using the SARS-COV array. After imputation data were exported in the standard PLINK format, downstream QC procedures and statistical analysis were conducted using the latest PLINK (http://pngu.mgh.harvard.edu/_purcell/plink) and R software packages (http://www.r-project.org/), installed on a Linux based computation resource. Specifically, imputation markers were kept if R2 > 0.8, and minor allele frequency (MAF) > 0.01. A HWE p-value <1 × 10^−20^ was used to control for potential genotyping clustering problems. A total of 12,457,719 SNPs were used for genome-wide analyses.

### Population genetic structure analysis

Genetic population structure was determined through PCA analysis after LD-pruning of associated markers. Briefly, after imputation, merging and QC filtering, genotype data were LD-pruned using plink and merged with 1000G samples from a Hg38 release (http://ftp.1000genomes.ebi.ac.uk/vol1/ftp/data_collections/1000_genomes_project/release/20190312_biallelic_SNV_and_INDEL/). PCA analysis was conducted both using plink and akt (https://github.com/Illumina/akt). Principal components used as covariates for GWAS analysis (see GWAS Analysis section) were derived without using genotype information from the 1000G samples.

### GWAS analysis

We used a dichotomous category defined among SaMi-Trop participants. As previously described, we have grouped individuals with a positive Chagas serology and any ECG alteration compatible with CCC into the group of individuals with CCC. Individuals with a positive *T cruzi* serology and no ECG alteration were classified as with the indeterminate form. A sensitivity analysis excluding individuals with mild ECG alterations and using only those with normal ECG versus those with major ECG alterations was also conducted. Baseline categorical parameters are presented using frequencies (proportions), continuous parameters are presented using mean ± SD.

Genome-wide association analyses were conducted using PLINK. We have conducted analyses adjusting for the first three principal components. Association analysis was conducted at the genotype level using an additive model. As sensitivity analysis we have also adjusted our additive model for sex and derived new models using dominant and recessive genetic models. The threshold for genome-wide significance was set to *p* <5×10^−8^. Associations with *p* <1×10^−6^ were considered as suggestive and presented as a list of candidate associated SNPs.

Local association plots were created using LocusZoom [12]. Local linkage disequilibrium structure was determined using Haploview [13].

### REDS II Sample, GWAS and fixed-effects meta-analysis

Details on the sample characteristics, genotyping array and CCC definition for the REDS II study have been published elsewhere [14]. Briefly, REDS-II was a retrospective cohort study, in which 499 *T*. *cruzi* seropositive (SP) blood donors (cases) identified by blood bank screening (255 from the city of São Paulo and 244 from the city of Montes Claros in the State of Minas Gerais). This sample was compared to 101 previously diagnosed cases of cardiomyopathy from the Heart Institute of University of Sao Paulo Medical School. From July 2008 to October 2010, all individuals (blood donors and patients with CCC) were characterized by demographic survey and by a health questionnaire and medical evaluation, including electrocardiogram (ECG), echocardiogram (Echo) and laboratory tests. The presence of CCC was determined by an expert panel composed of three Brazilian cardiologists based on the evaluation of clinical, laboratory, EKG and Echo findings. DNA samples from participants were extracted and sent to the Genomics Core Facility at UCSF for genotyping using the Affymetrix Axiom Genome-Wide Latino (Axiom GW LAT 1) array (Affymetrix, Santa Clara, CA) [15].

Here, because we have adjusted our models in SaMi-Trop using a slightly different model than primarily reported in the REDS II GWAS we have accessed genotype raw data and submitted these data to the same filtering, QC, imputation and association analysis as the one previously described for the SaMi-Trop samples. After derivation of GWAS summary statistics for the REDS II sample, we have derived joint association statistics for the two studies using a fixed-effect meta-analysis approach and was calculated using the plink–meta-analysis routine.

### PheWas analysis

After identifying snps that could be proxies of the associated haplotype, we have used MR-base through the R package *ieugwasr* to search for prior GWAS studies reporting significant levels of association between a number of different traits and the selected tag-SNP. MR-base is a database and analytical platform for Mendelian randomization [16]. It allows performing a Phenome-wide association study (PheWAS), which entails searching for the effects of a genetic variant across all publicly available datasets part of the database, as well as allows you to download summary statistics from publicly available GWAS to conduct genetic colocalization analysis. The MR-base database also describes the study associated with the results and the effect size and directionality. It differs from the GWAS catalog in which associations below the GWAS catalog reporting threshold (currently 1e-5) can also be explored. The used p value cut-off level for this phewas analysis was p <1×10^−3^.

### Colocalization analysis

For colocalization analysis we have defined a window spanning 400Kb centered at the most associated variant in the identified genome-wide significant locus. Information on all variants within this region was used for colocalization testing. We have used the R package *coloc* for colocalization analysis [17]. Briefly, all protein quantitative traits found to be associated with the same GWAS hit as CCC had their respectively summary statistics retrieved and sequentially tested for colocalization with the results obtained for CCC association. As reference LD structure we used 1000 genomes 2012 European LD matrix (our sample has approximately 80% European ancestry). We used a threshold of H4 (the posterior probability that a single causal variant, or haplotype, could explain the local association pattern of both tested traits) > 0.7 as evidence for significant colocalization [18].

### Genomic annotations and Hi-C data

We have annotated the newly identified CCC risk locus using Hg38 and the UCSC genome browser (https://genome.ucsc.edu/). Hi-C data from chromosomal 18 associated region was downloaded from http://3dgenome.fsm.northwestern.edu/.

### GTEx V8 tissue differential expression analysis and enrichment analysis

We conducted enrichment analysis to identify biological pathways associated with the genes responsible for the blood protein traits observed to also be associated with our risk locus. For each protein, we mapped the corresponding gene(s) using uniprot (https://www.uniprot.org/). We merged all genes retrieved into a gene set that then was used to conduct biological pathways enrichment analysis. This set of genes was submitted to an over-representation analysis using the pathways described in Gene Ontology, KEGG and Reactome. Selected pathways were those significantly enriched at an FDR < 0.05.

Additionally, we explored the downstream transcriptional consequences the identified genome-wide association region. For this, we used the index variants and conducted differential gene-expression analysis (using a dominant model) for all transcripts available in GTEx V.8 in all available tissues. After fitting models for our genotype groups, we retrieved all genes differentially expressed at p < 1 x 10^−3^ and conducted an enrichment pathway analysis (through an overrepresentation analysis as described above). Enrichment analyses were performed using the R packages clusterProfiler [19] and enrichplot.

## Results

The SaMi-Trop study was designed to identify and characterize risk factors for CCC development in the Brazilian population.

We summarize the demographic, clinical and laboratory characteristics of the 2,964 participants included in this analysis in Table 1 according to CCC presence. All participants with and without Chagas cardiomyopathy were Chagas disease patients defined by a positive T cruzi sorology.

**Table 1:** Demographic, clinical and laboratory characteristics of SaMi-Trop participants according to CCC.

	Total (n = 2964)	Without chagasic cardiomyopathy (n = 581)	With chagasic cardiomyopathy (n = 2383)	p value
		(19.6%)	(80.4%)	
**Age, years**	58.6 (17–98)	55.8 (18–88)	59.3 (17–98)	<0.0001
**Female sex**	1928 (65.1%)	422 (72.8%)	1506 (63.3%)	<0.0001
**Race**				
**Yellow**	55 (1.9%)	12 (2.1%)	43 (1.8%)	0.86
**White**	624 (21.3%)	124 (21.4%)	500 (21.2%)	
**Indigenous**	11 (0.4%)	1 (0.2%)	10 (0.4%)	
**Brown**	1737 (59.2%)	347 (59.9%)	1390 (59.1%)	
**Black**	506 (17.2%)	95 (16.4%)	411 (17.5%)	
**Smoking habit**				
**Never smoked more than 100 cigarettes**	54 (5.4%)	7 (3.7%)	47 (5.8%)	0.24
**Smoke some days, but not all**	153 (15.3%)	32 (16.8%)	121 (15.0%)	
**Smokes at least one pack of cigarettes every day**	555 (55.6%)	101 (53.2%)	454 (56.2%)	
**Smokes one or two packs of cigarettes every day**	200 (20.0%)	46 (24.2%)	154 (19.0%)	
**Smokes at least two packs of cigarettes every day**	36 (3.6%)	4 (2.1%)	32 (4.0%)	
**Clinical history**				
**Without diabetes**	2591 (88.9%)	506 (88.5%)	2085 (88.9%)	0.739
**Without high blood pressure**	1180 (40.0%)	301 (51.9%)	879 (37.0%)	<0.0001
**Without chronic kidney disease**	2655 (91.3%)	526 (91.2%)	2129 (91.4%)	0.79
**Without myocardial infarction**	2808 (95.3%)	568 (98.1%)	2240 (94.6%)	<0.0001
**Without Benznidazole use**	181 (30.8%)	37 (27.8%)	144 (31.4%)	0.549
**ECG result**				
**ECG Normal**	581 (19.6%)	581 (100%)		
**ECG Minor**	817 (27.6%)		817 (34.3%)	
**ECG Major**	1566 (52.8%)		1566 (65.7%)	
**Heart rate**	66.5 (21–188)	67.3 (50–105)	66.3 (21–188)	0.0006
**Overall p duration**	110.8 (8–192)	110.9 (34–192)	110.7 (8–190)	0.07
**Negative maj_q_wave_abn**	2679 (90.4%)	581 (100%)	2098 (88.0%)	<0.0001
**Negative maj_isolat_stt_abn**	2619 (88.4%)	581 (100%)	2038 (85.5%)	<0.0001
**Negative major_brd_comp**	2403 (81.1%)	581 (100%)	1822 (76.5%)	<0.0001
**Negative major_brd_bdase**	2701 (91.1%)	581 (100%)	2120 (88.9%)	<0.0001
**Negative major_biv**	2883 (97.3%)	581 (100%)	2302 (96,6%)	<0.0001
**Negative major_bre_comp**	2857 (96.4%)	581 (100%)	2276 (95.5%)	<0.0001
**Negative minor_essv**	2900 (97.8%)	581 (100%)	2319 (97.3%)	0.0001
**Negative major_mp**	2888 (97.4%)	581 (100%)	2307 (96.8%)	<0.0001
**Negative major_fa**	2852 (96.2%)	581 (100%)	2271 (95.3%)	<0.0001

As expected, the studied sample was highly admixed, especially between European and African ancestries (S1 Fig).

### Genome-Wide Association Analysis of Chagas Cardiomyopathy

We have performed a GWAS of the genetic association architecture of CCC. Groups were 581 individuals with a positive *T*. *cruzi* serology and no ECG abnormalities (Chagas indeterminate form) and 2,383 individuals classified as having signs of CCC. In the primary analysis, we have adjusted for the first 3 Principal Components correcting for the known population structure usually observed in studies using samples from the Brazilian population [20]. The observed genomic inflation factor for the adjusted analysis was lambda = 1.05.

**Fig 1:**
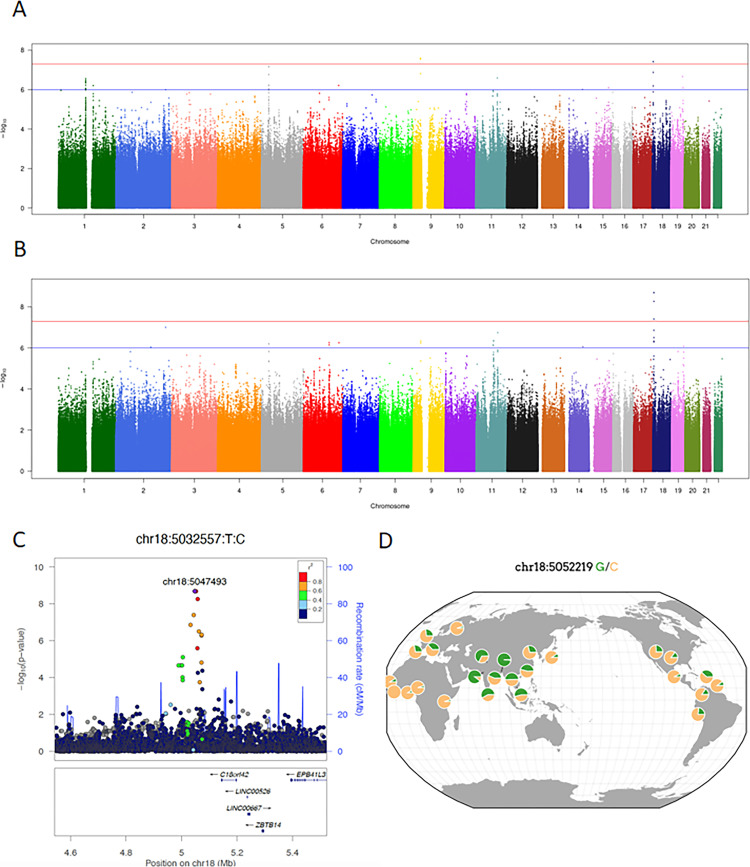
Overall results from SaMi-Trop and meta-analysis results. A. Manhattan plot of SaMi-Trop Study for Chagas cardiomyopathy. Results from a logistic regression model adjusted for the first 3 PCs. B. Manhattan plot of the fixed-effect meta-analysis results using SaMi-Trop and REDS II summary results. C. Local association plot of chromosome 18 genome-wide associated region. D. 1000G allele frequency distribution for rs34238187 (from https://popgen.uchicago.edu/ggv/). Allele frequencies normalized for 0.1 (green slices represent a percent of 0.1).

We observed two genome-wide significant loci that reached the pre-defined genome-wide significant level of 5 x 10^−8^ (Fig 1A). We also observed nine additional loci that reached the pre-defined p-value threshold of 1 x 10^−6^ (S1 Table and S2 Fig). We have run sensitivity analysis excluding individuals with minor ECG abnormalities from the analysis, using a dominant model or recessive model, and adjusting for sex as an additional covariate. Results were very similar to those observed in the main analysis (S3 Fig).

### Study of genome-wide significant hits in the REDS-II Study

In order to provide additional evidence of the newly observed genome-wide significant associations we have used data from a previous GWAS conducted by our group for Chagas cardiomyopathy. Before conducting the new association analysis, raw genotype data from the REDS II study were filtered and imputed using the same approach taken for the SaMi-Trop Study. In addition, we have adjusted the same model for the REDS II Study. At this stage we have only attempted to conduct association analysis using the two genome-wide significant hits using the index (most associated) snp in each loci. For the locus on *CYP4F26P* we did not observe any evidence of association in the REDS II study (rs62559910, OR 1.03, 95CI 0.76–1.40, pvalue 0.83). On the other hand, we observed a similar effect size and directionality in the association seen on *C18orf42* (rs34238187, OR 0.20, 95CI 0.06–0.69, pvalue = 0.01).

### SaMi-Trop and REDS II meta-analysis

After the new data processing of the REDS II data (imputation, QC filtering and new logistic regression model adjustment), we did not observe any candidate association region (S4 Fig, lambda 1.02).

Fixed-effect meta-analysis confirmed as the only genome-wide significant association signal the one local at chromosome 18 near *C18orf42* (Fig 1B).

In addition to the genome-wide significant hit at *C18orf42* we also identified 12 loci with associations at 10^−6^ or lower, presented at Table 2 (S5 Fig). All summary statistics for the fixed-effect meta-analysis can be accessed at Data Availability statement section.

**Table 2:** Index markers of genome-wide significant and suggestive loci associated with CCC.

CHR	BP	SNP	A1	N	P	OR	Q	NEAREST GENE	RSID
1	118760136	chr1:118760136:T:G	T	1	2,75E-07	0,7015	NA	TBX15	rs2764472
2	152591133	chr2:152591133:C:T	T	2	9,01E-07	0,4877	0,5691	FMNL2	rs185954378
2	216569920	chr2:216569920:C:T	T	2	9,88E-08	0,48	0,8933	RPL37A	rs6737722
5	32862370	chr5:32862370:A:G	G	2	6,31E-07	0,3645	0,0363	NPR3	rs115656580
6	112137404	chr6:112137404:T:C	C	2	5,54E-07	1,3974	0,5042	LAMA4	rs7764213
6	154277027	chr6:154277027:C:T	T	2	5,60E-07	0,4147	0,5128	IPCEF1	rs116800629
9	33595238	chr9:33595238:G:A	A	2	4,62E-07	1,5056	0,0182	CYP4F26P	rs10971593
11	76377183	chr11:76377183:T:C	C	2	4,50E-07	0,4809	0,5092	THAP12	rs115317331
11	94370564	chr11:94370564:C:T	T	2	1,78E-07	0,4867	0,3086	GPR83	rs11020751
14	80411016	chr14:80411016:T:G	G	2	8,57E-07	0,4619	0,3966	DIO2-AS1	rs17110631
**18**	**5052220**	**chr18:5052220:C:G**	**G**	**2**	**2,03E-09**	**0,3483**	**0,3608**	**C18orf42**	**rs34238187**
19	53972554	chr19:53972554:G:A	A	2	8,20E-07	0,7358	0,8796	CACNG8	rs12463378

BP are in Hg38

N—number of studies with data for marker (Sami-Trop or REDS-II)

Q–Cochran’s Q heterogeneity statistics

### Local association structure, haplotype association, and ancestry specific allele frequencies at the *C18orf42* locus

The newly identified genome-wide significant locus near *C18orf42* was characterized by two markers with the highest association signal chr18:5047494:C:T (rs34147216), and chr18:5052220:C:G (rs34238187), both with unadjusted p-values of 3.4 x 10^−9^ in the SaMi-Trop sample). As expected, they are in complete linkage disequilibrium (LD) in our sample with a MAF of 0.021 (the G allele from chr18:5052220 is protective for CCC). Using these two markers as index snps for our association and studying the local LD pattern in our sample we were able to refine the association region to a haplotype of approximately 30Kb spanning chromosome 18 from 5028302 to 5057621 (S6 Fig).

Using publicly available data and rs34238187 as the proxy snp for the associated haplotype, we retrieved the population-based frequency of our risk locus. In gnomAD v3.1.2, the overall allele frequency was 0.023. Among different datasets one consistent trend was the finding of a reduced allele frequency among African and African-American samples and a higher allele frequency among Central and South Asian samples (see Fig 1D for data from the 1KG worldwide distribution).

### rs34238187 is associated with several blood protein traits

We explored the association structure between rs34238187 and GTEx gene expression levels in all tissues and for all genes within the region. Results from GTEx cis-eQTL analysis did not retrieve any highly significant association between rs34238187 genotype and nearby gene expression levels.

Next, querying the MR-base database for other phenotypes previously shown to be associated with rs34238187, we observed 12 traits at a p-value cut-off of 10^−4^. Extending the p-value cut-off to 10^−3^, we observed additional 68 loci. Of particular interest, we observed several associations with blood protein levels, most of them inflammatory-related proteins (Table 3). All protein traits are associated with proteins coded by genes in distant genomic locations (*trans* pQTLs). Among the 9 blood protein traits observed when using the p-value cut-off of 10^−4^, all had strong genetic colocalization signals with CCC, suggesting no confounding by LD for these concordant associations (S2 Table).

**Table 3:** Significant associations between blood protein levels and rs34238187. Data from the INTERVAL study and retrieved through the mr-base database.

Trait	Study id	p-value	Gene(s)
E3 ubiquitin-protein ligase CCNB1IP1	prot-a-414	5,50E-06	CCNB1IP1
Ephrin-A5	prot-a-901	8,71E-06	EFNA5
Kunitz-type protease inhibitor 1	prot-a-2823	2,29E-05	SPINT1
Glycosyltransferase-like protein LARGE1	prot-a-1700	3,89E-05	LARGE1
Peroxisomal NADH pyrophosphatase NUDT12	prot-a-2126	4,07E-05	NUDT12
Ribosyldihydronicotinamide dehydrogenase [quinone]	prot-a-2084	4,90E-05	NQO2
Protein sel-1 homolog 2	prot-a-2664	5,13E-05	SEL1L2
Ras-related protein Rab-35	prot-a-2476	6,17E-05	RAB35
Linker for activation of T-cells family member 1	prot-a-1701	7,59E-05	LAT
Cardiotrophin-1	prot-a-711	1,66E-04	CTF1
Ephrin type-A receptor 2	prot-a-957	1,74E-04	EPHA2
Netrin receptor UNC5D	prot-a-3169	1,74E-04	UNC5D
Transcription factor RelB	prot-a-2516	1,78E-04	RELB
Dynactin-associated protein	prot-a-879	1,95E-04	DYNAP
Pyruvate kinase PKLR	prot-a-2282	2,14E-04	PKLR
Synaptic vesicle glycoprotein 2ª	prot-a-2905	2,14E-04	SV2A
Ephrin-B2	prot-a-904	2,19E-04	EFNB2
Cyclic AMP-responsive element-binding protein 3-like protein 4	prot-a-654	2,19E-04	CREB3L5
Inositol monophosphatase 3	prot-a-1548	2,34E-04	IMPAD1
Low-density lipoprotein receptor-related protein 8	prot-a-1780	2,51E-04	LRP8
Tyrosine-protein kinase receptor TYRO3	prot-a-3129	2,75E-04	TYRO3
Collagen alpha-3(VI) chain	prot-a-623	2,75E-04	COL6A3
Roundabout homolog 1	prot-a-2577	3,16E-04	ROBO1
Neuronal growth regulator 1	prot-a-2028	3,31E-04	NEGR1
Ectonucleoside triphosphate diphosphohydrolase 1	prot-a-950	3,31E-04	ENTPD1
Peptidyl-prolyl cis-trans isomerase FKBP2	prot-a-1114	3,63E-04	FKBP2
Legumain	prot-a-1734	3,72E-04	LGMN
Zinc finger protein 10	prot-a-3259	4,07E-04	ZNF10
C-reactive protein	ieu-b-4764	4,15E-04	CRP
Kallikrein-11	prot-a-1656	4,37E-04	KLK11
Ectodysplasin-A, secreted form	prot-a-886	4,47E-04	EDA
Roundabout homolog 2	prot-a-2578	4,57E-04	ROBO2
Toll-like receptor 4	prot-a-2990	4,79E-04	TLR4
Neurexophilin-1	prot-a-2132	4,90E-04	NXPH1
Protein kinase C-binding protein NELL2	prot-a-2031	5,01E-04	NELL2
Interleukin-1 receptor type 1	prot-a-1496	5,01E-04	IL1R2
Fms-related tyrosine kinase 3 ligand	prot-a-1128	5,01E-04	FLT3LG
SLIT and NTRK-like protein 5	prot-a-2769	5,01E-04	SLITRK5
Collagenase 3	prot-a-1913	5,13E-04	MMP13
Histone-lysine N-methyltransferase 2C	prot-a-1676	5,50E-04	KMT2C
Interleukin-36 gamma	prot-a-1528	5,62E-04	IL36G
C-C motif chemokine 3-like 1	prot-a-408	5,75E-04	CCL3L1, CCL3L3
Transmembrane glycoprotein NMB	prot-a-1255	5,89E-04	GPNMB
Leucine-rich repeats and immunoglobulin-like domains protein 3	prot-a-1770	6,17E-04	LRIG3
Kv channel-interacting protein 1	prot-a-1616	6,92E-04	KCNIP1
Poly [ADP-ribose] polymerase 1	prot-a-2182	7,08E-04	TNKS
Choline/ethanolamine kinase	prot-a-544	7,59E-04	CHKB
Serine/threonine-protein kinase 16	prot-a-2877	7,59E-04	STK16
Complement decay-accelerating factor	prot-a-453	7,59E-04	CD55
NADH dehydrogenase [ubiquinone] 1 beta subcomplex subunit 4	prot-a-2023	7,76E-04	NDUFB4
Transmembrane glycoprotein NMB	prot-a-1256	7,94E-04	GPNMB
P-selectin glycoprotein ligand 1	prot-a-2668	8,32E-04	SELPLG
Ephrin type-B receptor 2	prot-a-962	8,32E-04	EPHB2
Kallikrein-8	prot-a-1668	9,12E-04	KLK8
Ephrin type-B receptor 4	prot-a-964	9,33E-04	EPHB4
Dual specificity protein kinase CLK2	prot-a-586	9,55E-04	CLK2
Cytokine receptor-like factor 1:Cardiotrophin-like cytokine factor 1 Complex	prot-a-666	9,55E-04	CRLF1, CLCF1

Mapping of genes using protein name used the UniProt resouce (https://www.uniprot.org/)

### Gene Pathway Enrichment Analysis of associated blood protein traits show an over-representation of immune and inflammatory pathways

Using a p-value cut-off of 1 x 10^−3^, we selected all blood protein traits from Sun et al. that described pQTLs for the INTERVAL study retrieved from the MR-base database. From these, we mapped all proteins to their coding genes and used these as input for an over-representation analysis. From the 57 proteins, we mapped 59 genes (Table 3).

**Fig 2:**
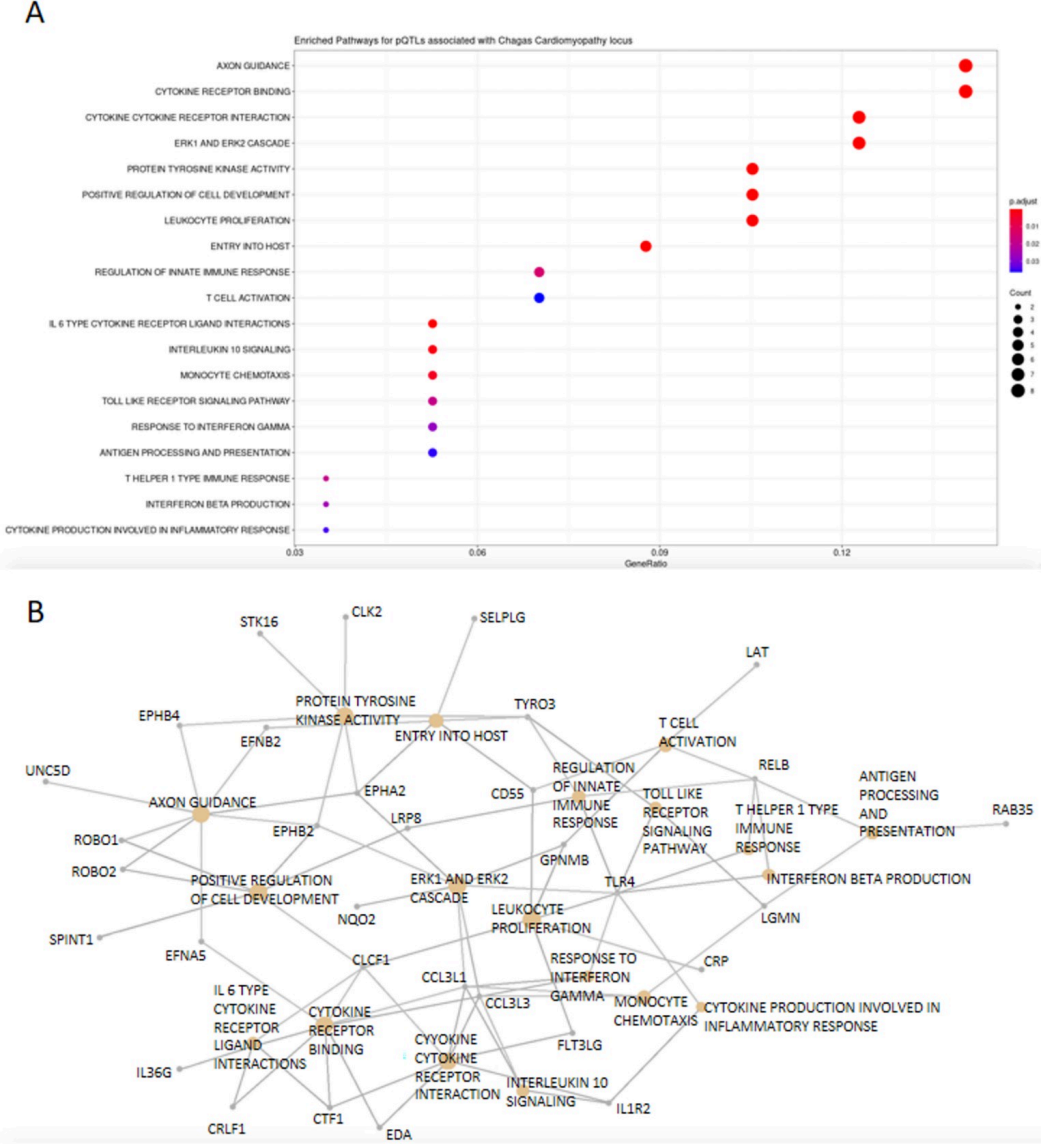
Over-representation analysis results of genes associated with blood proteins associated with rs34238187 on the INTERVAL study. A. Dotplot of selected enriched pathways. B. Network plot of the same selected enriched pathways as in A. Nodes (gray circle) are genes, hubs (orange circle) are selected pathways.

After restricting our analysis to pathways described only in Gene Ontology, KEGG, and Reactome, we observed 212 enriched pathways at an FDR level of 0.05. Several of these biological pathways are associated with inflammation, intracellular infection, innate immune response and interferon response, such as cytokine receptor binding (adjusted p-value = 0.03), ventricular system development (p = 2.3e-06), entry into host (p = 1.6e-04), leukocyte proliferation (p = 2.7e-04), regulation of innate immune response (p = 1.415530e-02) and T-helper 1 type immune response (p = 1.9e-02), details on S1 File and Fig 2.

### GTEx transcriptional analysis recapitulate pQTLs enrichment analysis results and suggest potential immune-mediation for newly observed genome-wide association with CCC

Studying the functional annotations associated with the chromosomal 18 30Kb haplotype (chr18:5028302 to chr18:5057621) we were not able to identify any significant genomic feature that could explain the observed association between the haplotype and inflammatory or immune-mediated pathways (S6 Fig). We reasoned that long-range genomic interactions might be responsible for long-range gene expression modulation by the risk haplotype that could lead to different transcriptional phenotypes. Thus, we have leveraged Hi-C data from a number of different cell types and tissues to better understand the long-range chromatin interactions occurring at this locus. S7 Fig shows that the identified minimal haplotype region interacts with several different genes 5’ from rs34238187 spanning a region of more than 10 Mb at chromosome 18. This interaction pattern was observed in several different cell types and tissues (S8B–S8D Fig).

Acknowledging that rs34238187 might be associated with transcriptional changes from an extended region, we hypothesized that the chromosome 18 haplotype tagged by rs34238187 could also be associated with transcriptional changes in the long-ranging interaction region defined by the Hi-C TAD domain. For testing this hypothesis, we have used normalized gene expression data from all GTEx V8 available tissues and contrasted samples carrying or not the identified risk allele at rs34238187. Using the region defined by the TAD domain observed from Hi-C data we sought to identify what transcripts in the region were associated with rs34238187 genotype. We observed a number of genes nominally associated with the associated genotype in different GTEx tissues (S3 Table). Albeit observing these associations, we did not identify any particularly strong eQTL for rs34238187 in the TAD region.

**Fig 3:**
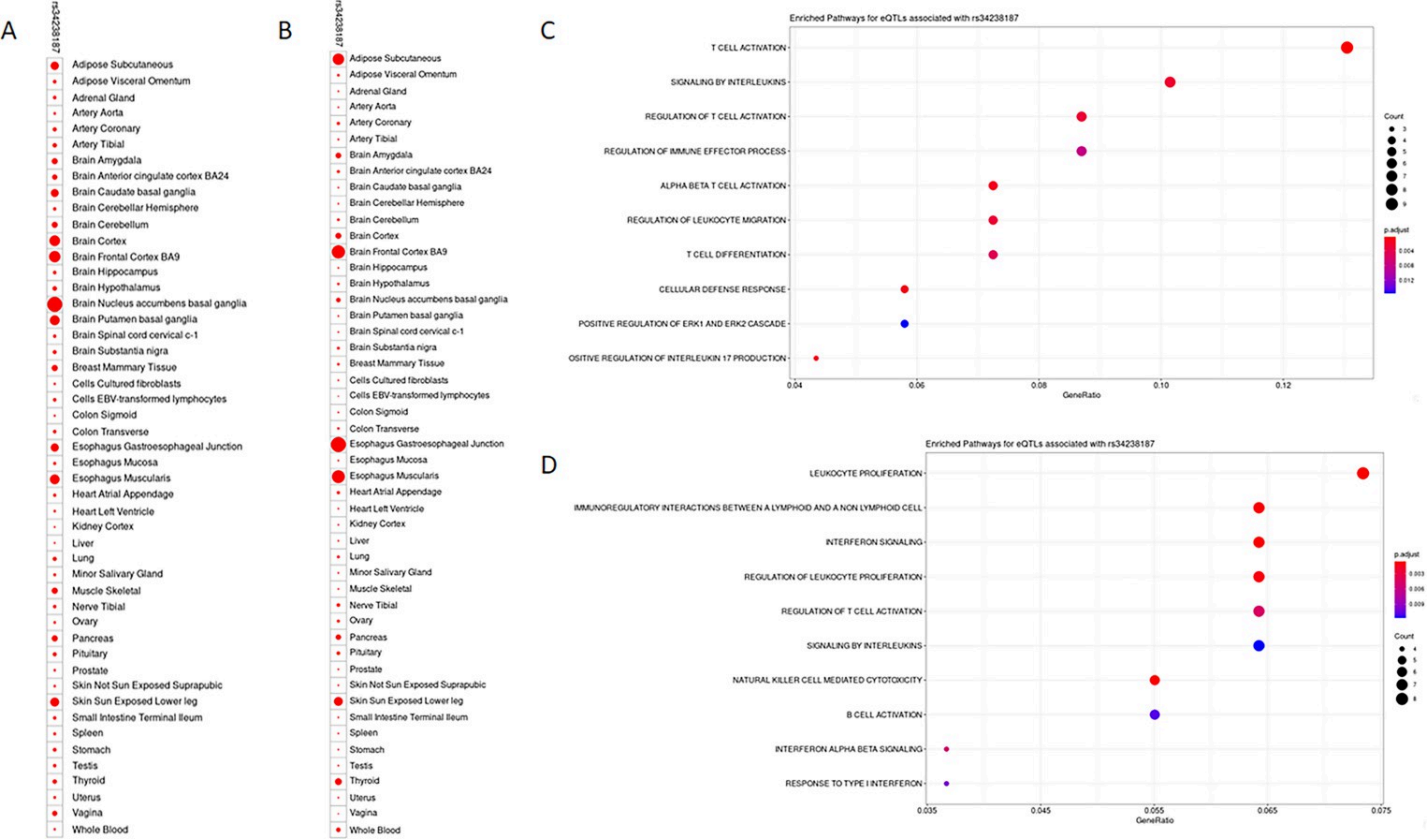
Results from GTEx V8 differential gene expression analysis and gene-pathway over-representation analysis. A. Dotplot of the number of genes with significant gene expression association with rs34238187 on different GTEx V8 tissues. B. Dotplot of the number og gene pathways with significant enrichment using DE genes from A. A significantly enriched pathway was defined as one with FDR p-value < 0.05 and at least two DE genes in the pathway. C. Selected significantly enriched pathways from DE genes observed in Adipose Subcutaneous tissue. D. Selected significantly enriched pathways from DE genes observed in Esophagus Muscularis tissue.

Finally, we hypothesized that rs34238187 could be, in GTEx tissues, associated with transcriptional changes similar to the protein changes observed in whole-blood using data from the INTERVAL study. For testing this, we retrieved the differential gene expression associated with rs34238187 (p < 1 x 10^−3^) in each tested tissue among all expressed genes in GTEx V8. We then used this set of differently expressed genes to conduct over-representation analysis on a per tissue basis. Fig 3 describes the number of differently expressed genes (Panel 3A) and significantly enriched gene-pathways (Panel 3B) on each of the tested GTEx tissues. A total of 1800 differently expressed genes and 1579 significantly enriched pathways were observed (all significantly enriched pathways can be retrieved in S2 File). Interestingly, the enrichment appeared to be more relevant for adipose, brain and esophagus tissues. In each of these highly enriched tissues, a similar picture could be observed where pathways associated with cytokine signaling, inflammation and immune modulation could be observed (Panels 3B, 3C and 3D).

## Discussion

Understanding the determinants of Chagas cardiomyopathy fulfills an urgent economical and social need. We have leveraged data from two of the largest studies to date on the genetic determinants of CCC, the SaMi-Trop cohort (6) study and the REDS II [21], and describe the first genome-wide significant risk allele for CCC which is also associated in a second, independent, sample. The newly identified locus, tagged by rs34238187 and located on chromosome 18 near *C18orf42* is also associated with a series of plasma blood protein traits, and a transcriptional profile, that is compatible with a role in immunity modulation, and thus, has a biological link with both Chagas disease and CCC development.

CCC is a mysterious disease. First, only 30% of individuals infected by the intracellular parasite Trypanosoma cruzi develop the disease and the data supporting the few variables suggested to predict CCC development come mostly from cross-sectional studies, making it very difficult to impute causality on these risk factors [14]. Secondly, despite the known role of humoral and cellular immunity in disease modulation [22], it is still unclear whether interindividual innate differences in these responses are able to explain disease development, or if they are just consequences or pleiotropic alterations seen during disease development [23]. In fact, in this specific scenario, the use of a genetics unbiased approach brings the potential to not only identify genetic variants associated with disease, but also to contribute to a better understanding of disease biology.

Through haplotype association analysis and linkage disequilibrium haplotype block determination we restricted the associated region to a 30Kb haplotype present in approximately 3% of the population. Data from a series of publicly available resources failed to provide functional evidence that the 30Kb haplotype region has a significantly active regulatory role regarding gene density or gene expression modulation. Nonetheless, Hi-C data from several different studies show that the 30Kb region is able to interact with other chromosome 18 regions as distant as 10Mb from rs34238187. To be sure, taking the large-range chromatin interaction regions we were able to observe significant associations between rs34238187 and transcript levels of several genes present in this extended chromosomal region.

Although we were not able to identify the specific causal genetic variant responsible for the association, or the specific gene, or genes, withing the associated genetic region that is being modulated by the 30Kb mapped haplotype, the newly identified locus is associated with a very distinct blood protein phenotype in participants from the INTERVAL study [24]. Interestingly, leveraging genome-wide transcriptional data from GTEx we were able to associate the newly identified risk haplotype with gene expression levels that are able to recapitulate the same observed enriched pathways as seen in the protein profile signature. It is also interesting to note that the three tissues where this was most evident are tissues known to be important in Chagas disease pathology: adipose tissue [25], brain [26] and esophagus [27].

Taken together, both protein and transcriptional profiles associated with the newly identified risk haplotype are enriched for cytokine and immune-related biological pathways, an association that is concordant to what is known about Chagas disease and CCC development from human [28–30] and animal [31,32] studies.

Heart tissue inflammation is the hallmark of CCC. Contrary to what is seen in the acute phase of Chagas disease, which is characterized by diffuse myocarditis with cardiomyocyte necrosis and intense inflammatory cell infiltration [33], in CCC there is persistent inflammation with the advent of fibrosis [34]. In the heart tissue of the carrier of CCC, the inflammatory infiltrate is disproportionate to the amount of parasite that persists in the heart and other tissues. In chronic Chagas patients, it has been demonstrated that there is an increased number of circulating activated T cells, which can secrete pro- and anti-inflammatory cytokines [35]. The cytokines produced by these activated lymphocytes regulate the immune response and are implicated in both resistance to infection and the clinical evolution of T. cruzi-infected individuals [36,37]. In fact, monocytes, macrophages, and the plasma of patients with the indeterminate form produce significantly higher amounts of IL-10 compared to individuals with CCC [38–41]. In contrast, pro-inflammatory cytokines, such as gamma interferon (IFN-γ), TNF, and IL-17, are at higher levels in TCD4, TCD8, and monocyte cells, in addition to serum and plasma from patients with CCC [42–45]. This association has been also replicated at the transcriptional level [46–47]. The expression of genes related to NK cell function is upregulated in patients with the indeterminate form, while they are downregulated in those patients with CCC. The association between our risk haplotype and a set of blood protein levels associated with inflammation and with innate and adaptive immunity suggest that these endophenotypes mediate the genotype association with CCC. As such, it is tempting to suggest that our GWAS data supports the role of genetically determined interindividual differences in the immune system response to intracellular pathogens and CCC development. These results warrant future studies aiming at the characterization of the functional consequences of the risk haplotype in patients with both CCC and indeterminate forms.

Despite the major economical and health importance of Chagas disease for Latin America, only two GWAS studies to date have been conducted to understand the genetic determinants of CCC. In 2013, Deng X et al. using data from the NHLBI Retrovirus Donor Study-II (REDS-II) described the genome-wide genetic association landscape for the disease for the first time [15]. The authors used a case-control design on 580 individuals and did not observe any genome-wide significant association with CCC. It is important to note the studies difference in relation to sample ascertainment, sample size and phenotype definition; all points that may have significant consequences in their final results. The REDSII Study, was conducted selecting controls from blood donation centers and cases from tertiary care cardiology institutions. It also has a smaller sample size (as compared to the Sami-Trop study). Sami-Trop has several advantages over previous GWAS studies on Chagas cardiomyopathy. It is a population-base study conducted within the geographical limits of an endemic area for Chagas disease, thus reducing potential bias due to geographical differences in *T cruzi* genotype and the possibility for reduced statistical power due to the interaction between host and pathogen genotypes. It has a significantly larger number of both cases and controls, thus providing increased statistical power. Cases are sampled from a continuous of severity, representing from the presence of only electrocardiographic abnormalities to severe left ventricle systolic dysfunction (REDSII cases were mainly those with severe left ventricle dysfunction). Despite the different designs and ascertainment schemes, the sample from both REDSII and Sami-Trop are similar regarding their genetic population structure, making the meta-analysis possible.

More recently, Casares-Marfil D et al., performed a cross-sectional, nested case-control study including samples from Colombia, Argentina, and Bolivia and meta-analyzed their results with the summary statistics reported by the REDS-II study [48]. The authors reported one statistically significant signal located in chromosome 11, rs2458298. In addition, two other suggestive loci were observed in the meta-analysis (rs10472156 and rs10759240). Results from the SaMi-Trop analysis did not replicate these associations with CCC in our sample (rs2458298, p-value = 0.41; rs10472156, p-value = 0.98; rs10759240, p-value = 0.41). Several possibilities may explain the discordant findings; differences in the population genetic structure between samples, the small sample size that might increase the chances of type I and II errors, differences in the clinical ascertainment and classification of samples between studies.

The main limitation of this study is the lack of the identification of a suitable candidate gene in the associated region. However, we show that the risk haplotype is able to interact with chromatin regions as far as 10 Mb from the index snp. In addition, the relatively small sample size may have precluded the identification of other genome-wide associated regions. The continuous effort to increase the sample size of the genetic studies for CCC are warranted, including the conduction of multi-country GWAS analysis in Latin America. In particular, future studies should strive to increase sampling of individuals with the indeterminate form, which are still under-represented in case-control studies of Chagas disease cardiomyopathy.

Another limitation is that we were not able to conduct stratified analysis by left ventricle systolic function. This could be potentially interesting to understand allele dose-response effects in chronic Chagas cardiomyopathy susceptibility. Finally, an intrinsic limitation of all cross-sectional analysis in Chagas disease that contrasts individuals with the indeterminate and chronic cardiomyopathy forms is the fact that individuals with the indeterminate form can convert to CCC even decades after the initial exposure [14]. In this regard, we have tried to balance both groups for age. However, even with all adjustments it is possible that the potential mis-assignment of participants to case and control groups contributes to reduce the statistical power of our study.

In conclusion, we identified a new risk genotype for CCC on human chromosome 18. Our analysis supports the hypothesis that the identified 30Kb haplotype may modulate CCC risk through relevant immunological endophenotypes associated with the risk haplotype both at the transcriptional and protein levels.

## Supporting information

S1 Fig Genetic Population Structure of SaMi-Trop samples. Panel A, plot using first principal component (PC1) versus second principal component (PC2). Panel B, plot using third principal component (PC3) versus second principal component (PC2). SAMI-TROP samples, black points. Other samples are from the 1000 Genomes project phase 3. EAS–East Asian, EUR–European, AMR–Amerindian, SAS–South Asian, AFR–African samples.
(TIF)

S2 Fig Local association plots of candidate loci on SaMi-Trop GWAS. (TIF)

S3 Fig Manhattan plot of Sensitivity Analysis. A. Excluding individuals with minor ECG abnormalities from the analysis; B. Using a dominant mode of action; C. Using a recessive mode of action; D. Adjusting for sex as an additional covariate. All models were adjusted for the 3 first principal components
(TIF)

S4 Fig Manhattan plot of new REDS II GWAS results for Chagas cardiomyopathy. No significant genomic inflation was observed (lambda = 1.02).
(TIF)

S5 Fig Local association plots of fixed-effect meta-analysis between SaMi-Trop and REDS II CCC GWAS results. (TIF)

S6 Fig Local Linkage Disequilibrium structure of chromosome 18 genome-wide significant locus. A. LD structure using the SaMi-Trop data spanning 200 Kb centered at rs34238187. Shown interval derived from genotype data from chr18:4952298 to chr18:5152024. B. LD structure of region with highest association signal, spanning from chr18:5028302 to chr18:5081267. C. Minimum haplotype region in complete LD with most associated snps. From chr18:5028302 to chr18:5057621.
(TIF)

S7 Fig Local annotation of chromosome 18 associated haplotype. Figure generated using the UCSC browser with Hg38 (https://genome.ucsc.edu/). Purple highlight minimum associated haplotype from chr18:5028302 to chr18:5057621. Note the lack of strong regulatory elements, as well as, the lack of coding genes spanning the associated haplotype region.
(TIF)

S8 Fig Profiling of chromatin conformation by HiC. Data obtained from http://3dgenome.fsm.northwestern.edu/. A. Upper panel data from "Rao, S. S. P., Huntley, M. H., Durand, N. C., Stamenova, E. K., Bochkov, I. D., Robinson, J. T. & Aiden, E. L. (2014). A 3D map of the human genome at kilobase resolution reveals principles of chromatin looping. Cell, 159(7), 1665–1680.". Lower panel Human genome assembly hg38. HiC resolution 40kb, chr18:2480000–7640000. Risk haplotype is from chr18:5028302 to chr18:5057621 and lies in a TAD domain encompassing from 4,000,000 to approximately 5,150,000. Other panels show same region using data from other tissues. B. Aorta (Leung, D., Jung, I., Rajagopal, N., Schmitt, A., Selvaraj, S., Lee, A. Y. & Ren, B. (2015). Integrative analysis of haplotype-resolved epigenomes across human tissues. Nature, 518(7539), 350–354.). C. HUVEC (Rao, S. S. P., Huntley, M. H., Durand, N. C., Stamenova, E. K., Bochkov, I. D., Robinson, J. T. & Aiden, E. L. (2014). A 3D map of the human genome at kilobase resolution reveals principles of chromatin looping. Cell, 159(7), 1665–1680.). D. Liver (Leung, D., Jung, I., Rajagopal, N., Schmitt, A., Selvaraj, S., Lee, A. Y. & Ren, B. (2015). Integrative analysis of haplotype-resolved epigenomes across human tissues. Nature, 518(7539), 350–354.).
(TIF)

S1 Table Loci that reached the pre-defined p-value threshold of 1 x 10^−6^ in SaMi-Trop GWAS. (XLS)

S2 Table Genetic colocalization PPH4 probability between blood protein traits and CCC. (XLSX)

S3 Table GTEx associations between genes and transcripts in the identified chromosome 18 TAD domain and rs34238187. (XLS)

S1 File Significantly enriched pathways among genes for associated blood proteins. (XLS)

S2 File Significantly enriched pathways in over-representation analysis of downstream DE genes associated with rs34238187 in different GTEx V8 tissues. (XLS)
