# Supplementary figures and images for: Genome-wide association study for Chagas Cardiomyopathy identify a new risk locus on chromosome 18 associated with an immune-related protein and transcriptional signature
Source: PLoS Negl Trop Dis. 2022 Oct 10;16(10):e0010725. doi: 10.1371/journal.pntd.0010725 (PMC9550069; doi:10.1371/journal.pntd.0010725)

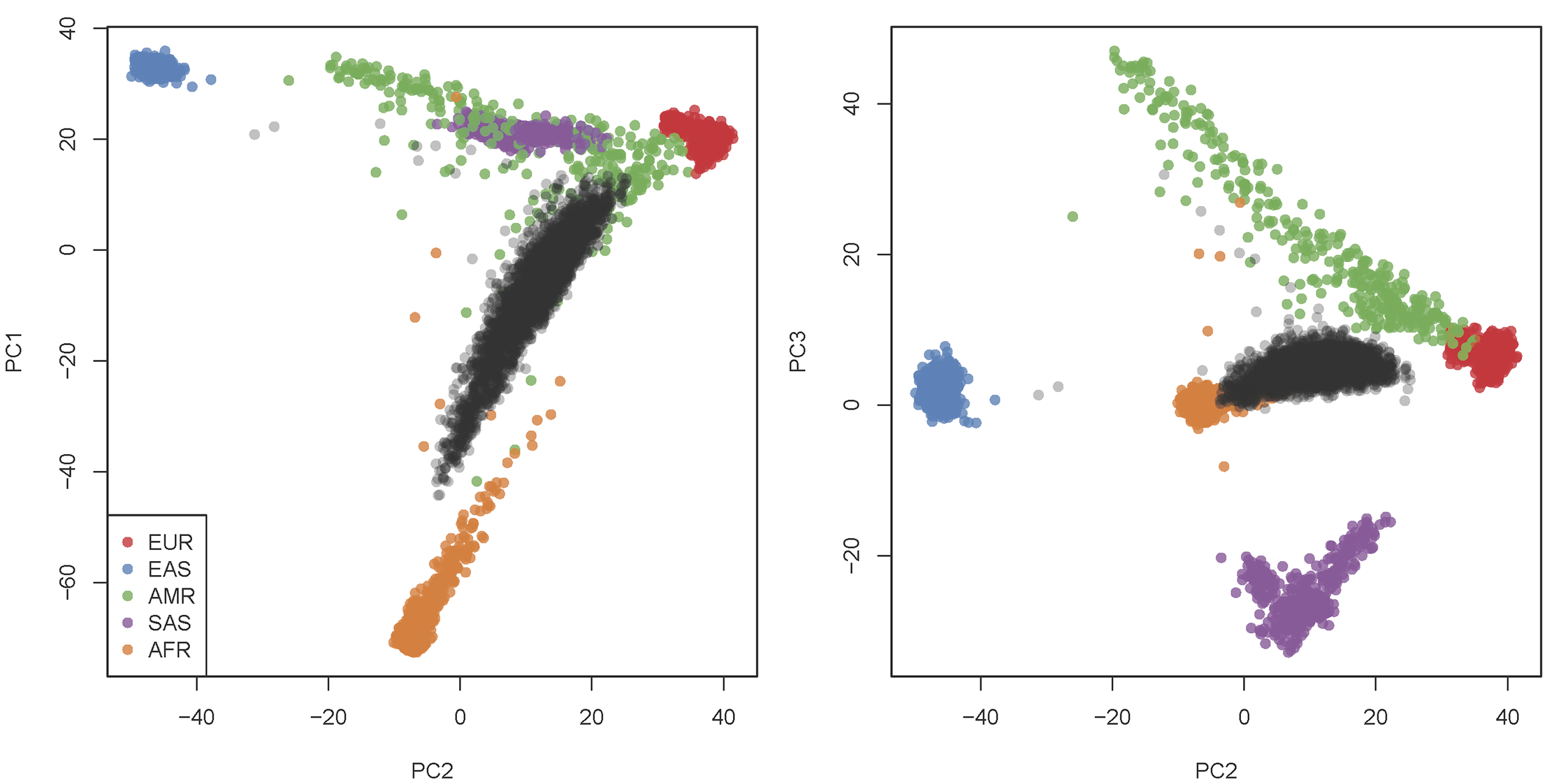

Supplement: S1 Fig — Panel A, plot using first principal component (PC1) versus second principal component (PC2). Panel B, plot using third principal component (PC3) versus second principal component (PC2). SAMI-TROP samples, black points. Other samples are from the 1000 Genomes project phase 3. EAS–East Asian, EUR–European, AMR–Amerindian, SAS–South Asian, AFR–African samples. (TIF) [file pntd.0010725.s001.tif]

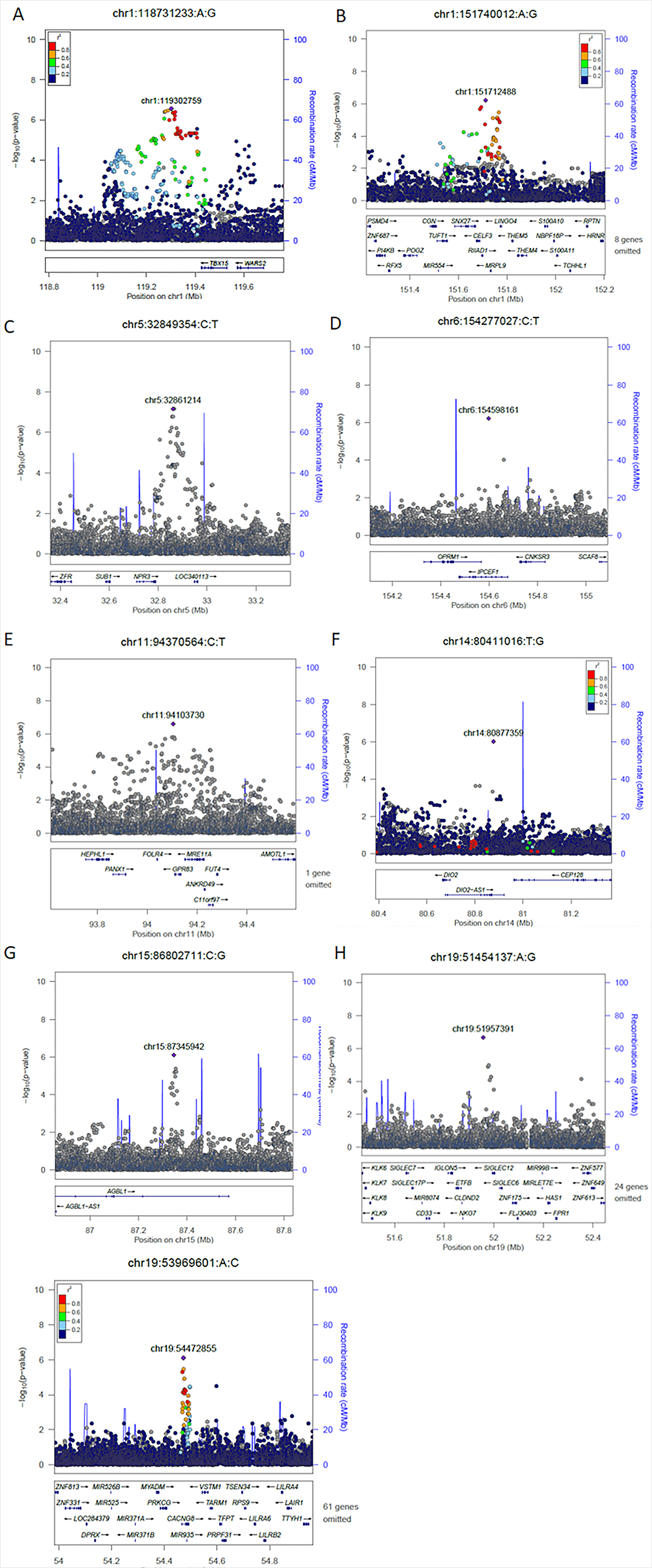

Supplement: S2 Fig — (TIF) [file pntd.0010725.s002.tif]

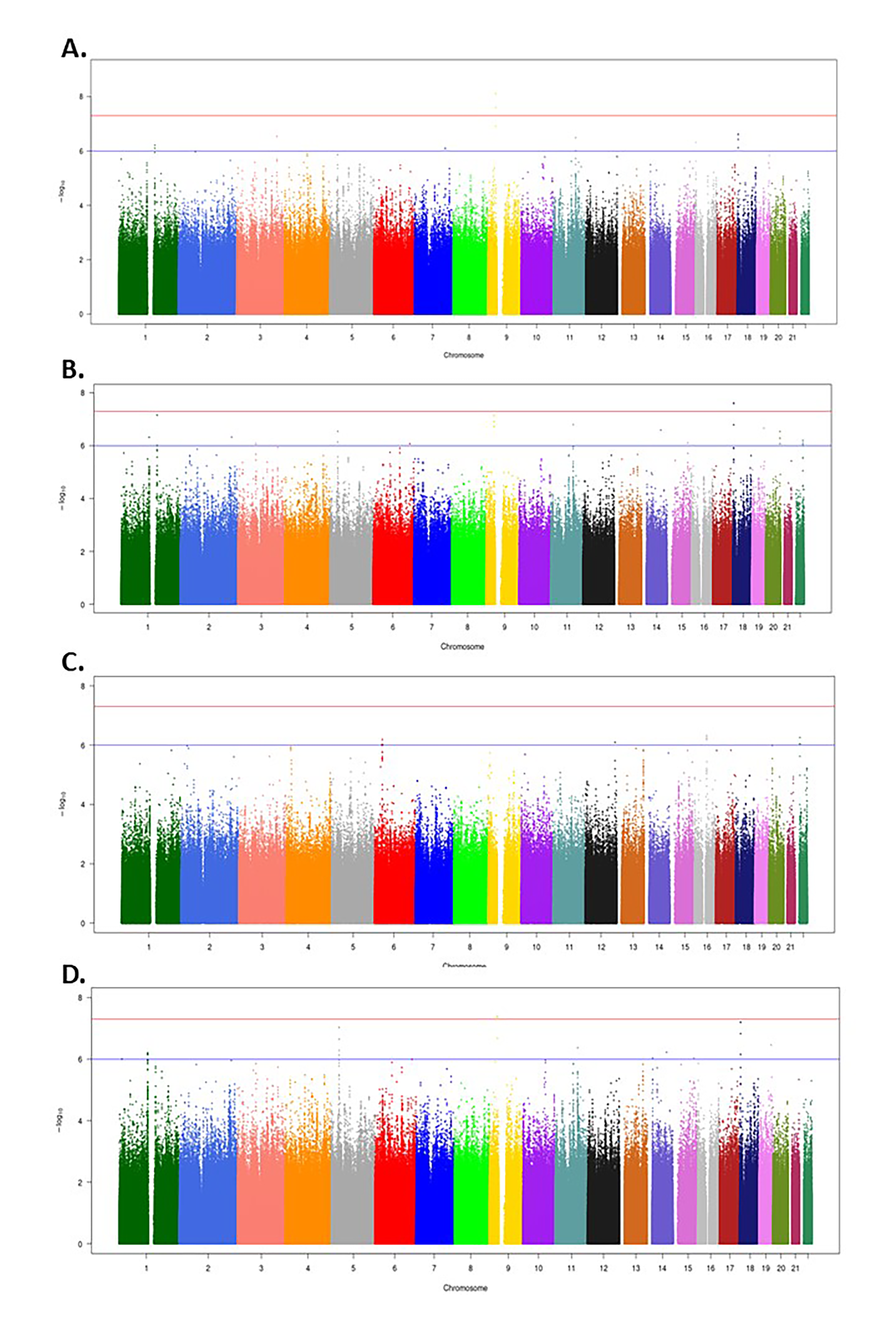

Supplement: S3 Fig — A. Excluding individuals with minor ECG abnormalities from the analysis; B. Using a dominant mode of action; C. Using a recessive mode of action; D. Adjusting for sex as an additional covariate. All models were adjusted for the 3 first principal components (TIF) [file pntd.0010725.s003.tif]

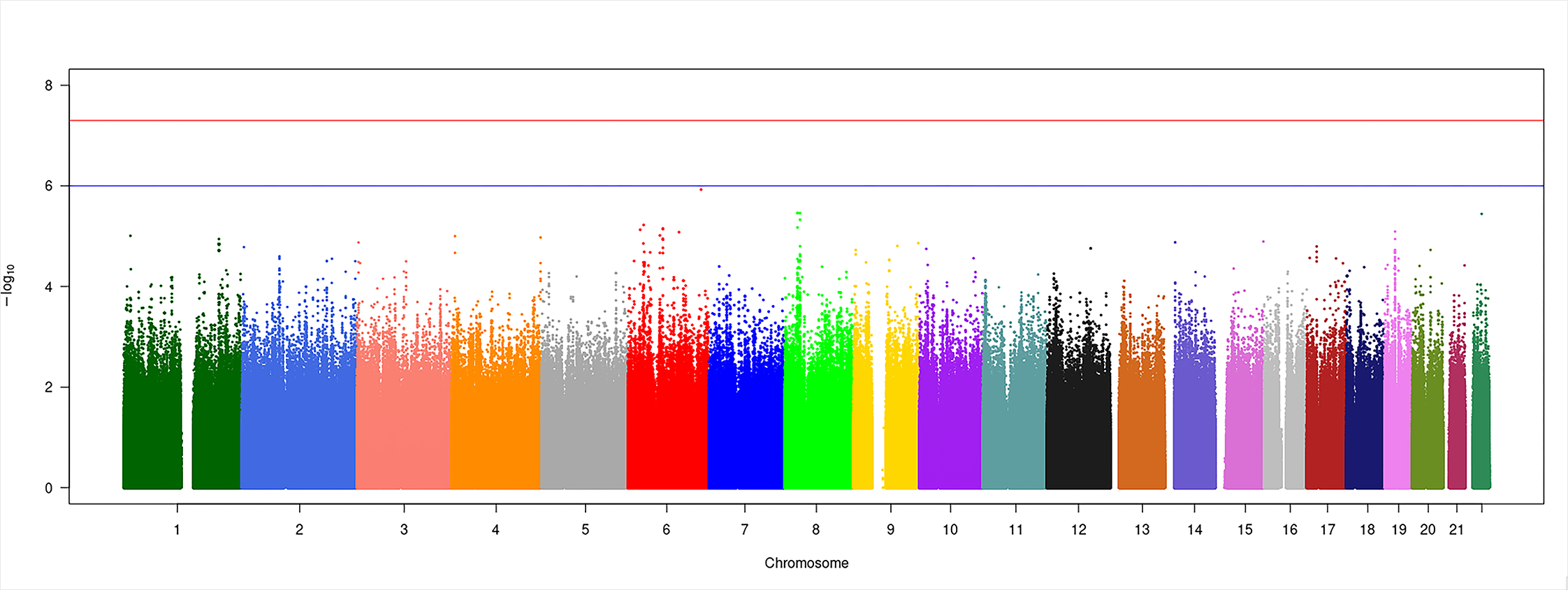

Supplement: S4 Fig — No significant genomic inflation was observed (lambda = 1.02). (TIF) [file pntd.0010725.s004.tif]

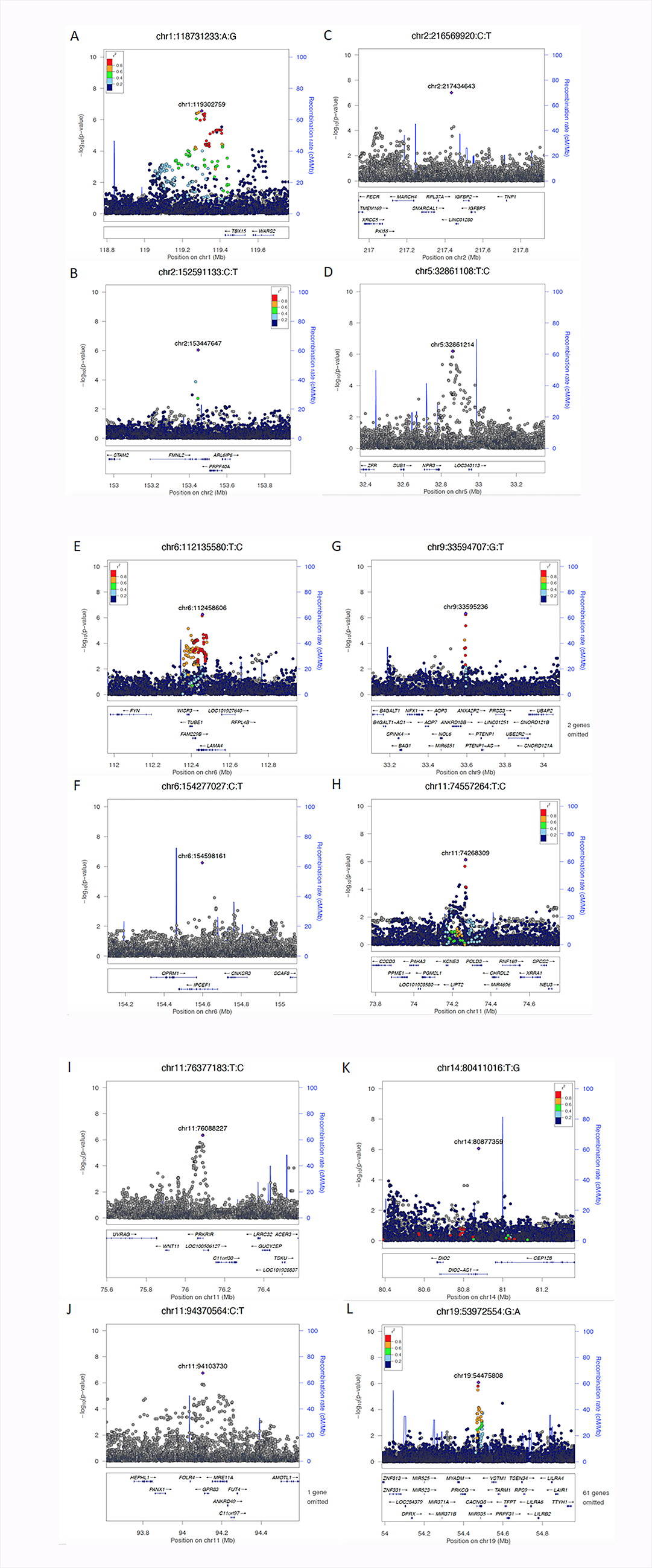

Supplement: S5 Fig — (TIF) [file pntd.0010725.s005.tif]

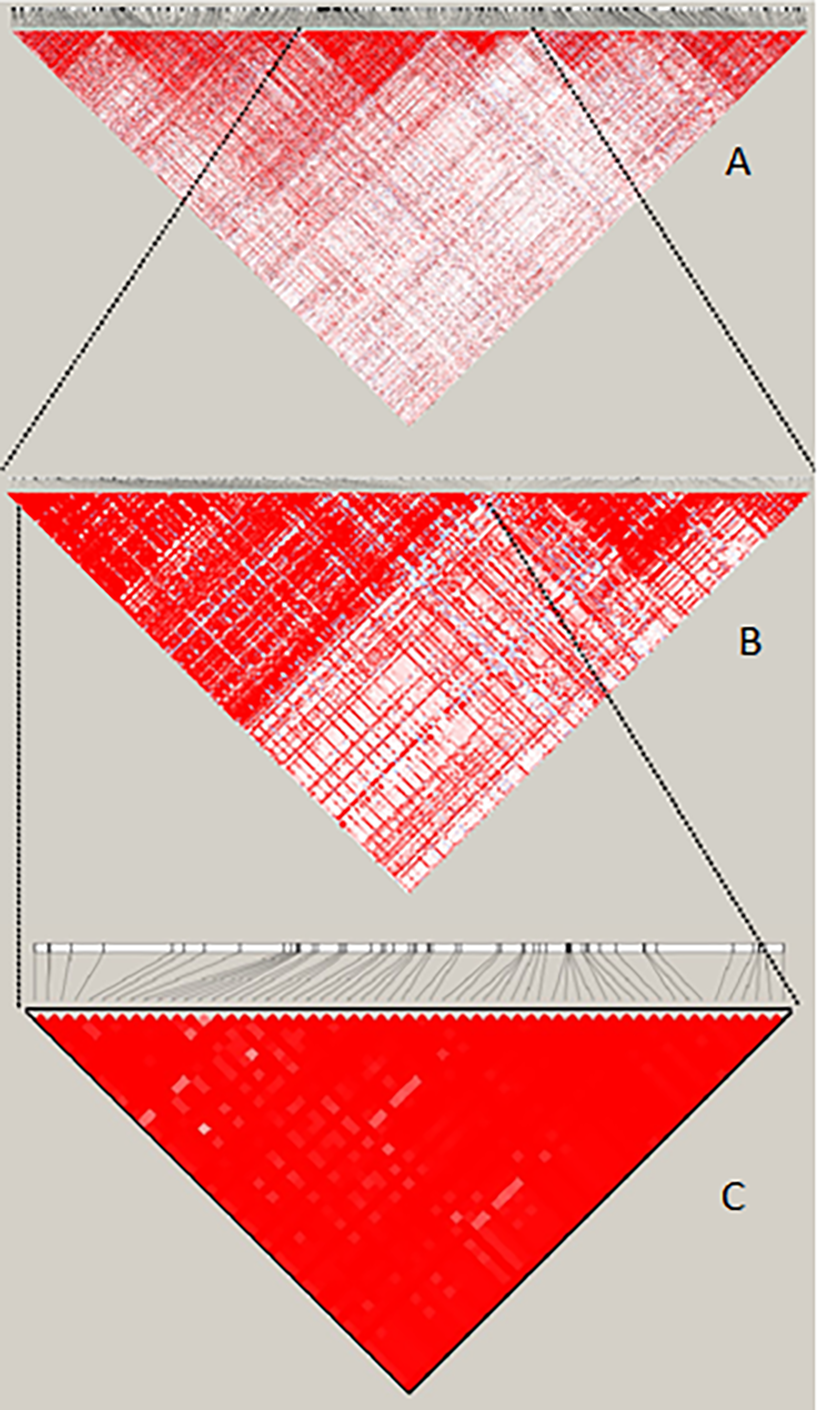

Supplement: S6 Fig — A. LD structure using the SaMi-Trop data spanning 200 Kb centered at rs34238187. Shown interval derived from genotype data from chr18:4952298 to chr18:5152024. B. LD structure of region with highest association signal, spanning from chr18:5028302 to chr18:5081267. C. Minimum haplotype region in complete LD with most associated snps. From chr18:5028302 to chr18:5057621. (TIF) [file pntd.0010725.s006.tif]

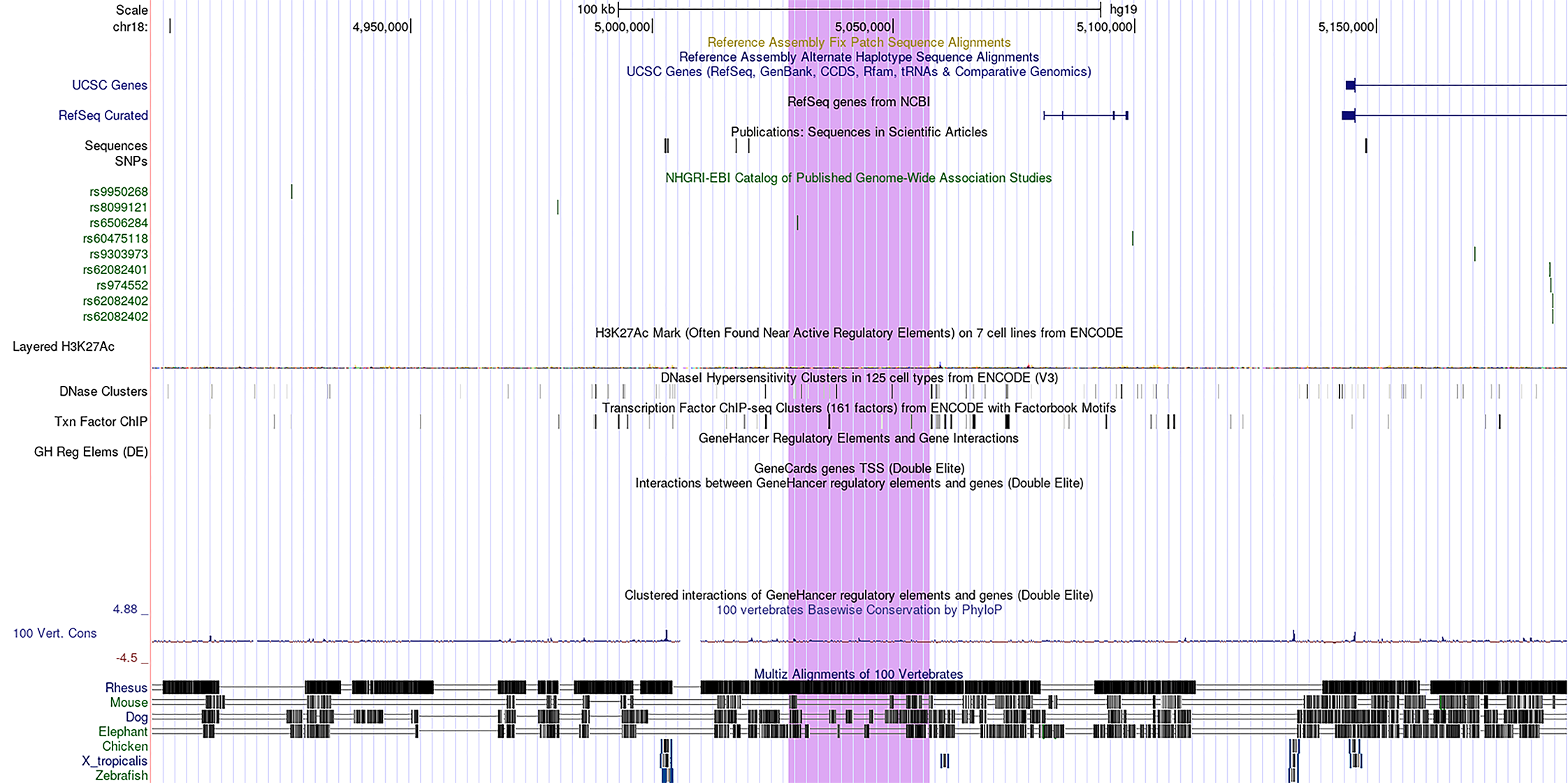

Supplement: S7 Fig — Figure generated using the UCSC browser with Hg38 (https://genome.ucsc.edu/). Purple highlight minimum associated haplotype from chr18:5028302 to chr18:5057621. Note the lack of strong regulatory elements, as well as, the lack of coding genes spanning the associated haplotype region. (TIF) [file pntd.0010725.s007.tif]

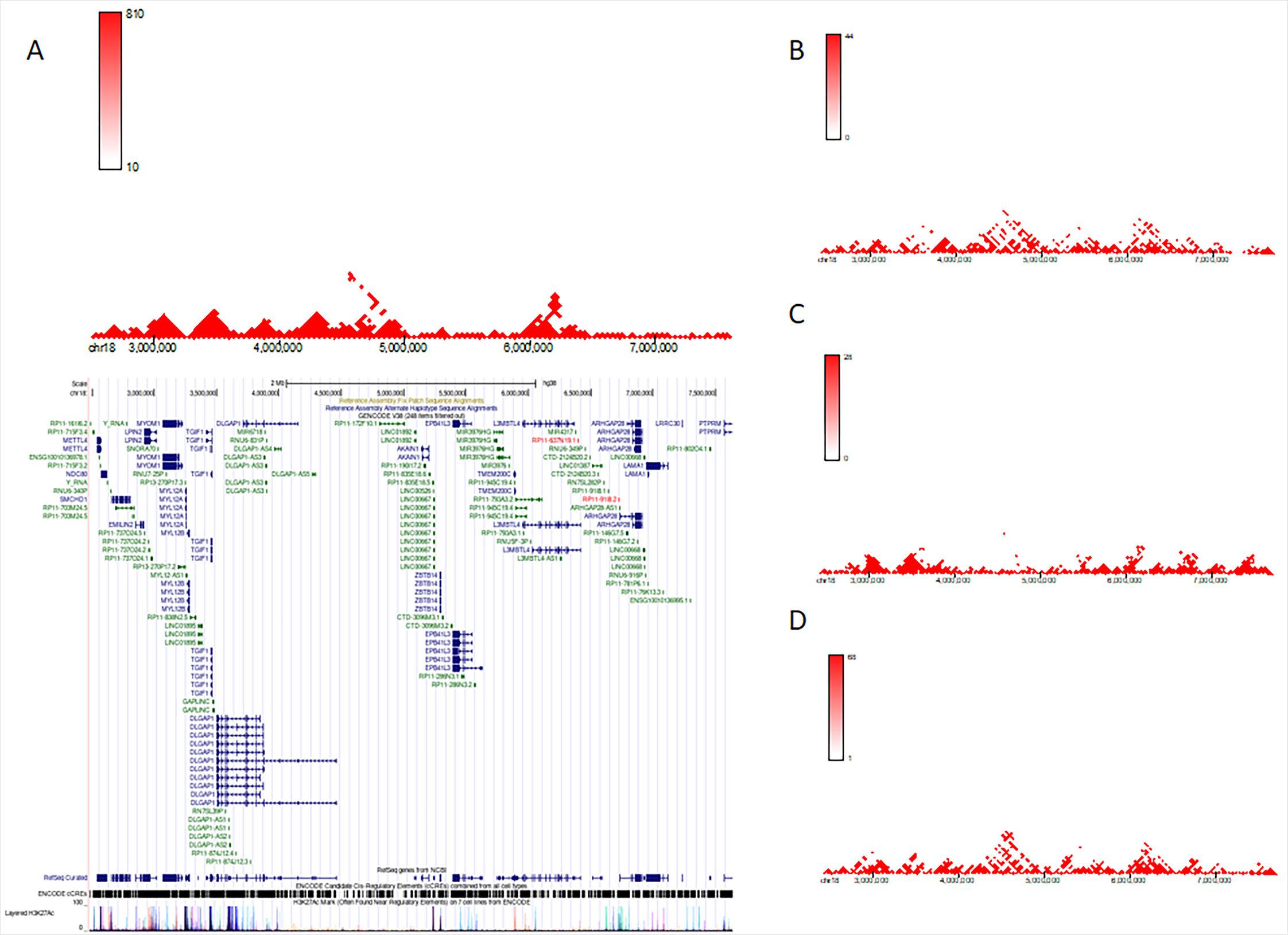

Supplement: S8 Fig — Data obtained from http://3dgenome.fsm.northwestern.edu/. A. Upper panel data from "Rao, S. S. P., Huntley, M. H., Durand, N. C., Stamenova, E. K., Bochkov, I. D., Robinson, J. T. & Aiden, E. L. (2014). A 3D map of the human genome at kilobase resolution reveals principles of chromatin looping. Cell, 159(7), 1665–1680.". Lower panel Human genome assembly hg38. HiC resolution 40kb, chr18:2480000–7640000. Risk haplotype is from chr18:5028302 to chr18:5057621 and lies in a TAD domain encompassing from 4,000,000 to approximately 5,150,000. Other panels show same region using data from other tissues. B. Aorta (Leung, D., Jung, I., Rajagopal, N., Schmitt, A., Selvaraj, S., Lee, A. Y. & Ren, B. (2015). Integrative analysis of haplotype-resolved epigenomes across human tissues. Nature, 518(7539), 350–354.). C. HUVEC (Rao, S. S. P., Huntley, M. H., Durand, N. C., Stamenova, E. K., Bochkov, I. D., Robinson, J. T. & Aiden, E. L. (2014). A 3D map of the human genome at kilobase resolution reveals principles of chromatin looping. Cell, 159(7), 1665–1680.). D. Liver (Leung, D., Jung, I., Rajagopal, N., Schmitt, A., Selvaraj, S., Lee, A. Y. & Ren, B. (2015). Integrative analysis of haplotype-resolved epigenomes across human tissues. Nature, 518(7539), 350–354.). (TIF) [file pntd.0010725.s008.tif]
